# Efficacy, Safety and Immunomodulatory Effect of Intramuscular Injections of Autologous Whole Blood Into Acupoints in Patients With Atopic Dermatitis: A Randomized Controlled Trial

**DOI:** 10.1002/iid3.70453

**Published:** 2026-04-20

**Authors:** Xuan Li, Xin‐ru Zhang, Ying‐qi Lin, Zhi‐qian Huang, Xin‐yan Li, Xing‐ru Yuan, Shu‐ping Liang, Yong Chen, Ying Wu, Yu‐ling Wang, Dong‐shu Zhang

**Affiliations:** ^1^ Department of Traditional Chinese Medicine, The Tenth Affiliated Hospital Southern Medical University (Dongguan People's Hospital) Guangdong China; ^2^ School of Traditional Chinese Medicine Southern Medical University Guangzhou China; ^3^ Department of Rheumatology and Immunology Affiliated Hospital of Zunyi Medical University Zunyi China; ^4^ Department of Biostatistics, School of Public Health Southern Medical University Guangzhou China; ^5^ Department of Dermatology, The Tenth Affiliated Hospital Southern Medical University (Dongguan People's Hospital) Guangdong China

**Keywords:** acupoint injection, atopic dermatitis, autologous whole blood, Dupilumab, immunomodulatory effect

## Abstract

**Introduction:**

The use of acupoint autohemotherapy (A‐AHT) has shown promise in the management of atopic dermatitis (AD); however, the lack of rigorously controlled comparative studies presents a significant obstacle to the establishment of strong clinical evidence.

**Aim:**

We aimed to compare the safety and efficacy of A‐AHT and subcutaneous Dupilumab injection in AD patients.

**Patients and Methods:**

Thirty‐one patients with moderate‐to‐severe AD were randomly allocated to receive 8 times of weekly intramuscular autologous whole blood (AWB) injection into bilateral Zusanli (ST36), Xuehai (SP10), Quchi (LI11) and Ashi points (*n* = 23) or subcutaneous Dupilumab injection with the dosage based on age/weigh (*n* = 8) for 8 weeks. Changes in SCORAD value, Visual Analog Scale (VAS) scores for pruritus and sleep quality, serum total IgE, cytokines including interleukin (IL)−2, IL‐4, IL‐6, IL‐10, interferon (IFN)‐γ, and tumor necrosis factor (TNF)‐α levels were assessed at baseline and week 8.

**Results:**

Both treatments resulted in a reduction of SCORAD values and serum IgE levels compared to their baseline (*p* < 0.001 or *p* < 0.05); however, no significant differences were observed between the two groups. When compared to the Dupilumab group, A‐AHT exhibited greater effectiveness in improving the VAS scores for pruritus and sleep quality. Within the A‐AHT group, there was a significant improvement in the serum levels of IL‐2, IL‐6, IL‐10, TNF‐α, and IFN‐γ at week 8 compared to baseline (*p* < 0.05 or *p* < 0.01), whereas no changes were noted in the Dupilumab group. Serious adverse events were not observed.

**Conclusions:**

A‐AHT appears to be effective in treating moderate‐to‐severe AD, warranting further studies to validate these results.

**Trail Registration:** ChiCTR2300068163.

## Graphical abstract

In a randomized trial of 31 moderate‐to‐severe atopic dermatitis patients, weekly intramuscular injection of autologous whole blood into bilateral acupoints (ST36, SP10, LI11, and Ashi; *n* = 23) was comparable to subcutaneous dupilumab injection (*n* = 8) over 8 weeks, improving SCORAD/IgE, with greater pruritus/sleep relief and broader cytokine suppression; treatment was well tolerated.

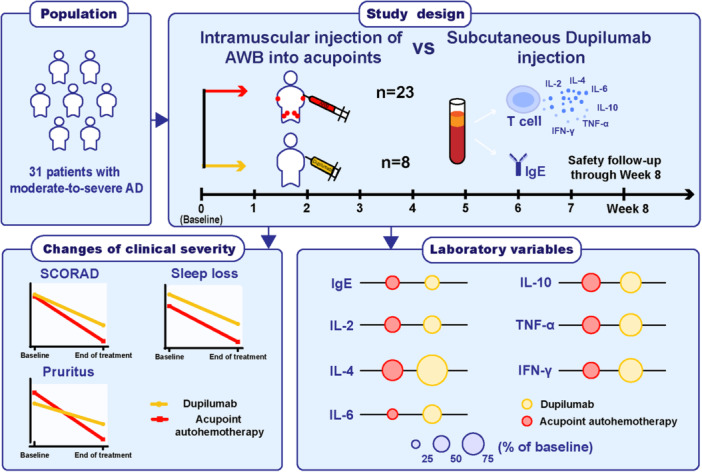

## 1 Introduction

Atopic dermatitis (AD) is a prevalent chronic inflammatory skin condition affecting up to 20% of individuals over their lifetime and significantly impacting quality of life. It is marked by severe itchiness, recurrent eczematous lesions, disrupted sleep patterns, and a fluctuating disease course [1, 2]. Management of AD remains largely symptom focused, with topical anti‐inflammatory therapies such as corticosteroids and/or calcineurin inhibitors used to provide relief. However, these therapies often yield disappointing clinical outcomes [3]. The current treatment options for AD offer only temporary relief during maintenance therapy and may not be effective for a subset of patients receiving dupilumab or JAK inhibitor [4]. Indeed, the high cost of dupilumab poses a significant economic burden for some patients. Consequently, there is a pressing need for the development of novel therapeutic strategies for AD patients.

Autohemotherapy, or autologous blood therapy (ABT), is a long‐standing medical treatment that involves the intramuscular administration of AWB‐ to address specific health conditions. This technique, first reported in 1913, has been employed for over a century by practitioners in Europe, America, and Asia to treat ailments such as AD and chronic urticaria [5, 6]. In China, leveraging traditional Chinese medicine (TCM) theory, a novel approach named A‐AHT has been developed, which combines autohemotherapy with acupuncture therapy and has been recognized as a representative project of intangible culture heritage. Our prior research showed promising therapeutic outcomes in a patient with refractory AD using A‐AHT [7]. Furthermore, our animal studies demonstrated that this method exerts immunomodulatory effects in BALB/c mice induced with 2,4‐dinitrochlorobenzene (DNCB) [8, 9, 10].

This study compared the clinical efficacy, safety, and immunomodulatory effects of A‐AHT versus dupilumab in patients with moderate‐to‐severe AD. To date, these interventions have not been directly compared in prior investigations.

## 2 Materials and Methods

### 2.1 General Information

**Figure 1:**
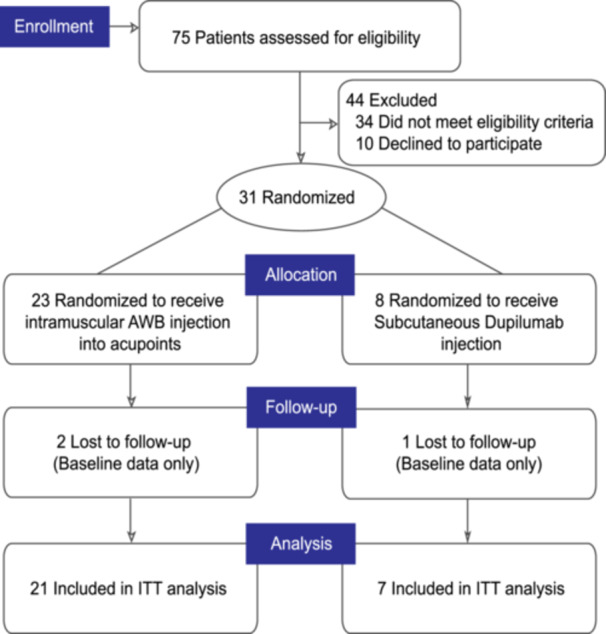
CONSORT flow diagram of the study. ITT, intention‐to‐treat.

This study was a single‐center, randomized, controlled trial (Register number: ChiCTR2300068163). The research was conducted at The Tenth Affiliated Hospital of Southern Medical University (Dongguan People's Hospital, Dongguan, Guangdong, China) from June 2024 to December 2024. Thirty‐one participants who met the inclusion criteria from the Outpatient Clinic participated in this study. After screening, there was a 7‐week intervention period (Figure 1).

The technique and design of the study adhered to Good Clinical Practice and the Declaration of Helsinki. Ethics approval was granted by the Institutional Review Board of Dongguan People's Hospital (KYKT2022‐04). All participants gave written informed consent.

### 2.2 Patient Selection and Eligibility Criteria

Eligible participants included infants, adolescents, and adults (aged ≥ 1.5 years) with a diagnosis of moderate‐to‐severe AD that had persisted for > 6 months and remained inadequately controlled despite topical corticosteroids and/or topical calcineurin inhibitors [11].

Participants were required to exhibit clinical features consistent with the established Classification Criteria for AD [12], a SCORing Atopic Dermatitis (SCORAD) score of 25 or higher (with the SCORAD scale ranging from 0 to 103, where elevated scores indicate increased clinical severity of AD [13]), and an affected body surface area of at least 10% at the initial screening and baseline.

Excluded from the study were those receiving systemic immunomodulatory therapy (e.g., corticosteroids, cyclosporine, or methotrexate) within the last 4 weeks, topical corticosteroids or topical calcineurin inhibitors users for 7 consecutive days before randomization. Additionally, patients with other active skin disorders that might interfere with study evaluations, pregnancy, lactation, severe psychological problems, concomitant severe systemic diseases, and hematologic or coagulation disorders were excluded from the study.

### 2.3 Randomization and Intervention

Block randomization with permuted blocks of four was generated by an independent statistician in R (v4.4.2; R Core Team, 2024; R Foundation for Statistical Computing, Vienna, Austria). To maintain allocation concealment, the randomization list was retained by a research coordinator not involved in participant contact. The list was secured in a locked file cabinet.

**Figure 2:**
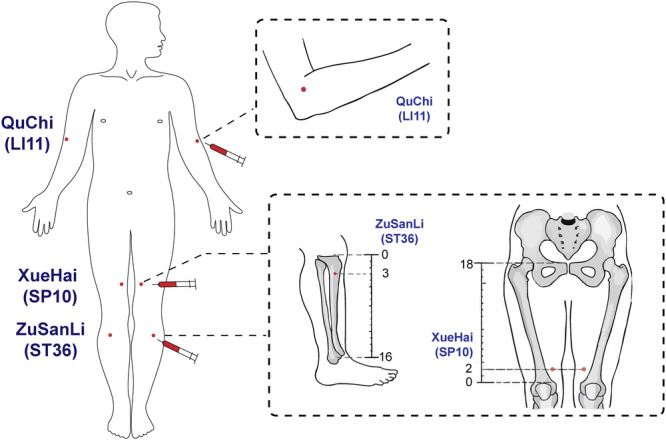
Diagram of A‐AHT and anatomical locations of injection sites. This diagram offers a visual depiction of the injection process and the placement of acupoints utilized in this method.

Patients were randomly assigned to receive either 8 times of weekly intramuscular injections of AWB into acupoints or subcutaneous injection of Dupilumab with the dosage and interval based on age/weight over a 7‐week intervention period (from baseline to week 7). The total doses of intramuscular injections of AWB were determined by age of the patients (5 mL for patients aged < 6 years; 10 mL for patients aged ≥ 6 years). At each session, from the patient's median cubital vein, whole venous blood was drawn to a total volume of 5 to 10 mL. Collection was performed with a 5‐ or 10‐mL syringe fitted with a 7# hypodermic needle (0.7 mm in diameter), and no heparin was used. Subsequently, the AWB was injected into bilateral Zusanli (ST36), Xuehai (SP10), Quchi (LI11), and Ashi point (the site of severe skin lesions) at a volume of 0.5–1.5 mL per acupoint, with larger volumes generally used at sites with thicker musculature [14] (Figure 2). A perpendicular needle insertion was performed to reach the intramuscular layer (2–3 cm deep), and it often elicited soreness or tightening at the injection sites. Completion of the procedure required no more than 3 min. Following the injection, patients were instructed to rest for 5 min to monitor for any discomfort. During the study period, all medications and procedures intended for AD treatment were discontinued, except for topical moisturizers (Vitamin E cream or petroleum jelly cream). Conversely, patients in the Dupilumab group received subcutaneous injection of dupilumab with the dosage tailored to the age and weight (Supporting Information Table S1). Additionally, these patients also received concomitant topical corticosteroids (TCS) and oral antihistamine.

Quchi (LI11): Located at the midpoint of the line connecting Chize (LU5) to the lateral epicondyle of the humerus; with the elbow flexed, this point lies at the lateral end of the transverse elbow crease.

Zusanli (ST36): Located at 3 cun below Dubi (ST35), one finger‐width from the tibia's anterior crest.

Xuehai (SP10): Located at 2 cun above the patella's medial superior border, on the bulge of the quadriceps femoris at its medial portion.

### 2.4 Primary Outcome Measures

The efficacy endpoints comprised AD clinical severity changes from baseline through week 8, evaluated using the following parameters, including the SCORAD score [13]., with elevated scores reflecting a more severe clinical manifestation of AD; visual analogue scale (VAS) for pruritus; VAS for sleep quality, VAS for overall skin condition, and body surface area affected (%). These clinical severity scores (SCORAD, VAS for pruritus, VAS for quality of sleep, VAS for overall skin condition and body surface area affected) were assessed weekly from week 0 (baseline) to weeks 4 and 8.

### 2.5 Secondary Outcome Measures

Venous blood samples were obtained at week 0, and weeks 8. Serum samples were stored in −20°C. Serum concentrations of IL‐2, IL‐4, IL‐6, IL‐10, TNF‐α, and IFN‐γ were quantified using enzyme‐linked immunosorbent assay kits based on monoclonal antibodies (Tianjin Kuangbo Tongsheng Biotechnology Co. Ltd, Tianjin, CHINA). The serum total immunoglobulin (Ig) E level was measured by chemiluminescence immunoassay using IMMULITE 2000 Total IgE (Siemens Healthcare Diagnostics Products Limited, Llanberis, UK).

### 2.6 Safety Assessment

Safety was assessed as the recording of adverse events from the initial injection through the final administration during an 8‐week observation period. The patients were questioned according to a special schema regarding pain, local reactions, skin manifestations, and general symptoms (Table 4).

### 2.7 Statistical Analyses

Efficacy was analyzed using the intention‐to‐treat population. The ITT population included all randomized participants with at least one administration of the study intervention. Categorical outcomes were compared with the chi‐squared test. When expected counts were < 5 in more than 20% of contingency‐table cells, Fisher's exact test was applied instead. Continuous outcomes were modeled with a mixed‐effects repeated‐measures approach, and least‐squares (LS) means were derived. The model included fixed effects for treatment, week, and treatment‐by‐week interaction, with a random intercept for individual subjects to account for between‐subject variability. Group differences were summarized as model‐based least‐squares mean changes from baseline to week 8 with 95% confidence intervals (CI) [15]. Week‐8 missing outcomes attributable to rescue treatment, withdrawal of informed consent, occurrence of adverse events (AEs), or insufficient therapeutic response were handled as nonresponders in the efficacy analysis of the study intervention. For laboratory biomarker assays, the small proportion of missing results (0.2%) arising from samples exceeding the linear range of the detection method was addressed by multiple imputation.

Within‐group changes in laboratory parameters were assessed using the Wilcoxon signed‐rank test, and between‐group differences were evaluated with the Mann–Whitney U test. All tests were two‐sided, with statistical significance defined as *p* < 0.05. Analyses were performed in R (version 4.4.2; R Core Team, 2024; R Foundation for Statistical Computing, Vienna, Austria).

## 3 Results

### 3.1 Study Population

By our calculation utilizing the Two‐Sample T‐Test Power Analysis, we determined that enrolling a total of 28 patients, with 21 allocated to the A‐AHT group and 7 to the Dupilumab group, would provide approximately 90% power for the trial to detect a statistically significant difference between the two study groups from baseline to week 8.

Allowing for an anticipated 10% dropout rate, we planned to enroll 23 patients in the A‐AHT group and 8 patients in the dupilumab group.

A total of 31 patients aged 1.5–58 years were randomized to receive either weekly intramuscular injections of AWB into bilateral acupoints (Zusanli, Xuehai, and Quchi) (*n* = 23) or subcutaneous injection of Dupilumab with the dosage based on age/weight (*n* = 8) at the baseline visit (Figure 1). The clinical and laboratory characteristics at baseline were comparable between the A‐AHT group and the Dupilumab group (Table 1).

**Table 1:** Clinical and laboratory characteristics at baseline.

Characteristic	All patients			Infant and adolescent patients		
	Dupilumab (*n* = 8)	A‐AHT (*n* = 23)	*p* value	Dupilumab (*n* = 7)	A‐AHT (*n* = 6)	*p* value
Age (year)	11.5 (1.5–40.0)	29.0 (9.0–58.0)	0.005	9.0 (1.5–16.0)	13.5 (9.0–15.0)	0.373
Male, sex	6 (75.0)	9 (39.1)	0.113	5 (71.4)	5 (83.3)	> 0.999
Duration of disease (year)	3.0 (1.0–20.0)	5.0 (0.5–30.0)	0.441	3.0 (1.0–10.0)	5.0 (1.5–6.0)	0.529
SCORAD score	69.15 (58.0–84.5)	61.7 (42.0–101.4)	0.291	63.4 (58.0–84.5)	51.5 (43.0–77.2)	0.138
Objective SCORAD score	55.0 (45.0–68.5)	45.7 (31.0–81.40)	0.311	51.4 (45.0–68.5)	40.5 (31.0–67.2)	0.138
VAS for pruritus	8 (3–8)	8 (4–10)	0.212	8 (3–8)	7.5 (6–8)	> 0.999
VAS for sleep loss	8 (6–10)	6 (2–10)	0.023	8 (6–9)	4 (2–6)	0.002
Body surface area affected (%)	50 (43–85)	40 (10–99)	0.572	52 (43–85)	43 (15–63)	0.469
Concomitant atopic diseases						
Allergic rhinitis	2 (25.0)	8 (34.8)	> 0.999	2 (28.6)	3 (50.0)	0.592
Allergic conjunctivitis	1 (12.5)	1 (4.3)	0.456	1 (14.3)	0 (0)	> 0.999
Asthma	0 (0)	2 (8.7)	> 0.999	0 (0)	0 (0)	
Laboratory parameters						
igE (pg/mL)	384 (100–2136)	363 (62–3625)	0.707	400 (100‐2136)	554 (62‐3625)	> 0.999

*Note:* Data are presented as number (%) or medians (range).

Abbreviations: A‐AHT, acupoint autohemotherapy; SCORAD, SCORing atopic dermatitis; VAS, visual analogue scale, Ig, immunoglobulin.

Overall, our sample included 6 infant and adolescent patients in the A‐AHT group (median age = 13.5) and a total of 7 infant and adolescent patients in Dupilumab group (median age = 9). However, patients in Dupilumab had significantly higher baseline VAS scores for quality of sleep than did infant and adolescent patients within A‐AHT group.

### 3.2 Clinical Efficacy Outcomes

There were statistically significant changes in the SCORAD score from baseline to week 4 and 8 in the A‐AHT group (*p* < 0.001) and the Dupilumab group (*p* < 0.001). However, there was no significant difference between the two groups from week 0 to week 4 and 8. The average percentage change in SCORAD between baseline and week 8 was −73.4% (95% CI, −86.5 to −60.3) in the A‐AHT group, −49.0% (95% CI, −70.9 to −27.2) in the Dupilumab group and −54.8% (95% CI, −76.4 to −33.2) in the infant and adolescent patients among the A‐AHT group.

**Figure 3:**
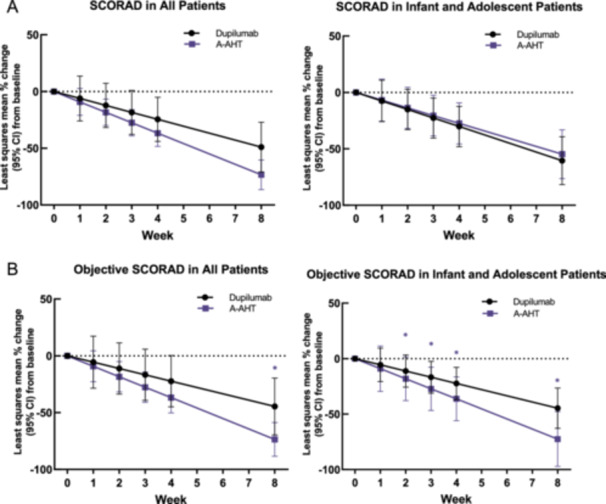
Changes in atopic dermatitis severity, shown as SCORAD (A) and objective SCORAD (B). Error bars represent 95% confidence intervals (CI). A‐AHT, acupoint autohemotherapy. * *p* < 0.05.

There was a statistically significant change in the objective SCORAD score from baseline to week 8 in the A‐AHT group (*p* < 0.001) and the Dupilumab group (*p* < 0.001). Additionally, there was a significant difference between the two groups at week 8 (*p* = 0.044). Furthermore, in infant and adolescent patients, there was significantly more improvement in objective SCORAD score at week 2, 3, 4, and 8 with AWB administration versus Dupilumab (*p* = 0.037, 0.026, 0.020, or 0.020). The average percentage change in objective SCORAD between baseline and week 8 was −73.6% (95% CI, −88.5 to −58.8) in the A‐AHT group, −44.6% (95% CI, −69.6 to −19.6) in the Dupilumab group and −72.5% (95% CI, −97.1 to −47.9) in the infant and adolescent patients among the A‐AHT group (Table 2 and Figure 3B).

**Table 2:** Changes in clinical severity parameters of atopic dermatitis.

Continuous parameters	Dupilumab infant and adolescent patients (*n* = 7)			A‐AHT all patients (*n* = 21)					A‐AHT infant and adolescent patients (*n* = 6)				
	Baseline, mean (SD)	Week 8, mean (SD)	Percentage change from baseline to week 8, LS mean (95% CI)	Baseline, mean(SD)	Week 8, mean (SD)	Percentage change from baseline to week 8, LS mean (95% CI)	Difference^1^ in percentage change, LS mean (95% CI)	*p* ^1^ value	Baseline, mean (SD)	Week 8, mean (SD)	Percentage change from baseline to week 8, LS mean (95% CI)	Difference^2^ in percentage change, LS mean (95% CI)	*p* ^2^ value
SCORAD score	59.9 (15.7)	30.5 (16.9)	−49.0% (−70.9 to −27.2)	57.9 (15.3)	15.4 (16.5)	−73.4% (−86.5 to −60.3)	21.3% (2.7 to 39.9)	0.051	56.2 (22.2)	25.4 (24.7)	−54.8% (−76.4 to −33.2)	−7.7% (−40.6 to 25.1)	0.548
Objective SCORAD score	46.5 (14.0)	25.7 (15.0)	−44.6% (−69.6 to −19.6)	45.2 (13.7)	11.9 (14.6)	−73.6% (−88.5 to −58.8)	27.6% (7.3 to 47.9)	0.044	37.3 (16.3)	10.2 (18.7)	−72.5% (−97.1 to −47.9)	23.8% (−4.5 to 52.0)	0.020
VAS for pruritus	5.8 (1.4)	3.5 (1.6)	−40.3% (−61.5 to −19.2)	7.0 (1.3)	1.8 (1.5)	−74.7% (−84.8 to −64.5)	29.9% (7.6 to 52.2)	0.021	6.4 (2.0)	3.3 (2.4)	−49.2% (−67.9 to −30.5)	−3.2% (−37.3 to 30.9)	0.372
VAS for sleep loss	7.0 (1.3)	3.7 (1.5)	−47.5% (−64.2 to −30.7)	5.7 (1.3)	1.7 (1.5)	−70.4% (−82.2 to −58.6)	19.1% (−5.6 to 43.8)	0.006	4.9 (3.4)	2.6 (3.6)	−45.9% (−82.2 to −9.6)	−16.8% (−70.0 to 36.4)	0.941
Body surface area affected (%)	51.7 (18.0)	21.3 (18.7)	−58.7% (−86.7 to −30.7)	45.4 (18.0)	12.6 (18.8)	−72.2% (−91.2 to −53.3)	6.0% (−21.3 to 33.2)	0.299	41.3 (24.9)	15.2 (26.5)	−63.2% (−94.7 to −31.6)	−5.4% (−26.6 to 15.8)	0.934

*Note:* For continuous variables, *p* values were obtained from a mixed‐effects model for repeated measures (MMRM) estimating LS means. *p* ^1^ denotes the between‐group comparison (dupilumab vs A‐AHT) of the percentage change from baseline to week 8 in the overall population. *p* ^2^ denotes the same comparison for the infant and adolescent subgroup.

Abbreviations: CI, confidence interval; LS, least squares; SCORAD, SCORing Atopic Dermatitis; SD, standard deviation; VAS, visual analogue scale.

**Figure 4:**
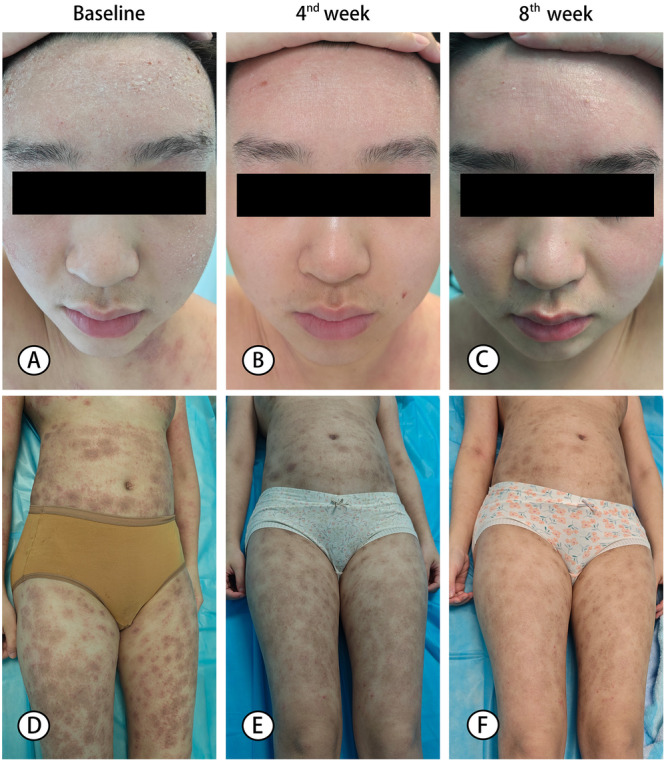
Clinical photographs of two patients with severe atopic dermatitis treated with A‐AHT weekly for 8 weeks. Patient 1: pretreatment baseline (A), week 4 (B), and week 8 (C). Patient 2: pretreatment baseline (D), week 4 (E), and week 8 (F).

There were statistically significant changes of VAS for pruritus, and VAS for quality of sleep between the A‐AHT group and Dupilumab group (*p* < 0.05 or *p* < 0.01). Nevertheless, there were no significant differences in the changes of body surface area affected (%) and overall skin condition between the two groups (*p* > 0.05) (Table 2). Marked improvements in overall skin appearance were evident in photographs from two patients following A‐AHT administration, consistent with sustained clinical benefit (Figure 4).

### 3.3 Changes in the Serum Levels of Total IgE

At baseline and week 8, there were no statistically significant differences in serum total IgE levels between the A‐AHT group and the Dupilumab group (*p* > 0.05) (Table 3). However, within both groups, there were significant changes in total IgE levels from baseline to week 8 (*p* < 0.001 or *p* < 0.05, respectively) (Table 3).

**Table 3:** Changes in laboratory parameters.

Laboratory characteristics	Dupilumab infant and adolescent patients (*n* = 7)		A‐AHT all patients (*n* = 21)			A‐AHT infant and adolescent patients (*n* = 6)		
	Median (range)	*p* value^a^	Median (range)	*p* value^a^	Inter‐group *p* ^1^ value^b^	Median (range)	*p* value^a^	Inter‐group *p* ^2^ value^b^
igE (pg/mL)								
Baseline	400 (100–2136)		363 (62–3625)		0.640	554 (62–3625)		> 0.999
Week 8	264 (21–1393)	0.016	298 (12–1366)	< 0.001	0.140	467.5 (19–1366)	0.094	0.731
IL‐2 (pg/mL)								
Baseline	1.29 (0–1.95)		1.1 (0–3.80)		0.928	1.195 (0.89–3.80)		0.534
Week 8	0.84 (0–1.80)	0.563	0.89 (0–2.00)	0.030	0.887	1.12 (0.56–1.41)	0.219	> 0.999
IL‐4(pg/mL)								
Baseline	1.32 (0.61–4.79)		1.98 (0.77–3.67)		0.909	2.03 (0.99–3.67)		0.628
Week 8	2.04 (0.78–8.01)	0.375	1.82 (0.29–2.85)	0.500	0.204	1.92 (1.62–2.67)	0.688	0.628
IL‐6(pg/mL)								
Baseline	1.69 (1.01–4.94)		2.24 (0.88–138.43)		0.499	2.54 (2.00–3.27)		0.731
Week 8	1.87 (0.76–6.32)	0.813	2.29 (1.00–47.89)	0.018	0.748	2.265 (2.00–2.64)	0.063	> 0.999
IL‐10(pg/mL)								
Baseline	1.72 (0.72–3.80)		2.94 (1.40–7.48)		0.048	3.215 (1.86–6.84)		0.073
Week 8	1.25 (0.97–3.03)	0.813	2.49 (1.30–5.12)	0.026	0.272	2.885 (1.71–4.62)	0.031	0.051
TNF‐α (pg/mL)								
Baseline	2.07 (0.52–3.73)		3.29 (0.67–11.14)		0.056	4.10 (3.29–11.14)		0.008
Week 8	2.20 (0.78–3.66)	> 0.999	2.31 (0.56–9.38)	0.030	0.172	2.875 (1.00–9.38)	0.031	0.181
IFN‐γ (pg/mL)								
Baseline	1.51 (0–4.16)		2.94 (0–5.80)		0.072	3.22 (2.32–4.65)		0.072
Week 8	1.64 (0.08–4.17)	0.688	2.00 (0–4.03)	< 0.001	0.056	2.81 (1.40–3.85)	0.031	0.073

*Note: p* ^1^ denotes the between‐group comparison (Dupilumab vs A‐AHT) in the percentage change from baseline to week 8 for the overall population. *p* ^2^ denotes the same comparison for infant and adolescent patients.

Abbreviations: A‐AHT, acupoint autohemotherapy; Ig, immunoglobulin; IL, interleukin‐10; TNF, tumor necrosis factor; IFN, interferon.

^a^ The *p*‐value for within‐group difference of the comparison with baseline (week 0) was analyzed by the Wilcoxon signed‐rank test.

^b^ The *p*‐value for inter‐group comparison was analyzed by the Mann‐Whitney U test.

In infant and adolescent patients, serum total IgE levels did not differ significantly between A‐AHT and Dupilumab at baseline or week 8 (*p* > 0.05). (Table 3). Additionally, within the infant and adolescent patients of A‐AHT group, there was no significant change in total IgE levels from baseline to week 8 (*p* > 0.05) (Table 3).

### 3.4 Changes in T Helper (Th)1, Th2, and Regulatory T Cells (Treg) Cytokine Serum Levels

At baseline, no statistically significant differences were observed in the serum levels of IL‐2, IL‐4, IL‐6, IL‐10, and IFN‐γ between the A‐AHT group and the Dupilumab group (*p* > 0.05), with exception of significant differences in serum TNF‐α level between the A‐AHT group and the dupilumab group among infant and adolescent patients (Table 3). Within the A‐AHT group, there was a significant improvement in the serum levels of IL‐2, IL‐6, IL‐10, TNF‐α, and IFN‐γ at week 8 compared to baseline (*p* < 0.05 or *p* < 0.01) (Table 3). Conversely, in the Dupilumab group, there was no notable change in the cytokine serum levels from baseline to week 8.

Among infant and adolescent patients, within the A‐AHT group, there was a significant decrease in the serum levels of IL‐10, TNF‐α, and IFN‐γ at week 8 compared to baseline (*p* < 0.05) (Table 3).

### 3.5 Side‐Effects

Serious adverse events were not reported. Among randomized patients, 42.9% (12/28) developed one or more adverse events (Table 4). The most frequently reported adverse event was reaction at the injection‐site, reported in 23.8% (5/21) of patients in the A‐AHT group and 28.6% (2/7) in the Dupilumab group (*p* > 0.999). Also commonly reported were exacerbation of AD, which were reported in 4.8% (1/21) of patients in the A‐AHT group and 42.9% (3/7) in the Dupilumab group (*p* = 0.038). Despite AD exacerbations in some patients, no patients in the A‐AHT group used systemicor topical corticosteroids during the course of the study.

**Table 4:** Adverse events.

Adverse events	Dupilumab (*n* = 7)	A‐AHT (*n* = 21)	*p* value
Atopic dermatitis exacerbation	3 (42.9)	1 (4.8)	0.038
Dizziness	0	0	
Pale complexion	0	1 (4.8)	> 0.999
Nausea and vomiting	0	0	
Syncope or shock	0	0	
Reaction at the injection site (including hematoma, pain, etc.)	2 (28.6)	5 (23.8)	> 0.999
Patients with 1 ≥ adverse event	0	0	
Patients with an adverse event leading to withdrawal from intervention	0	0	
Total number of serious adverse events	5 (71.4)	7 (33.3)	0.103

*Note:* Data are presented as *n* (%).

Abbreviation: AHT, acupoint autohemotherapy.

## 4 Discussion

To our knowledge, this was the first randomized clinical study to evaluate the clinical efficacy of A‐AHT compared with dupilumab. The current investigation found that A‐AHT demonstrated equivalent and significant clinical improvement compared to Dupilumab, marked by a reduction in objective clinical severity scores (SCORAD and objective SCORAD) and a decrease in serum total IgE levels among patients with moderate‐to‐severe AD. Compared to the Dupilumab group, the A‐AHT exhibited greater effectiveness in improving the VAS scores for pruritus and sleep quality. Notably, within the A‐AHT group, there was a significant improvement in the serum levels of IL‐2, IL‐6, IL‐10, TNF‐α, and IFN‐γ at week 8 compared to baseline, whereas no such changes were observed in the Dupilumab group. Additionally, a higher incidence of atopic dermatitis (AD) exacerbation was recorded in the Dupilumab group compared to the A‐AHT group. Importantly, no severe adverse events were reported following A‐AHT in AD patients. These results suggest the promising potential and safety of this intervention for the clinical management of AD.

Immune dysfunction in AD is characterized by elevated total serum IgE levels [16], overexpression of IL‐10 by Tregs [17], and a significant enrichment of Th1, Th2, and Th17 immune responses. Successful modulation of atopic dermatitis therefore requires the down‐regulation of Th1/Th17 and Th2 type of immune responses [18]. Recent research has demonstrated that Chinese patients with moderate‐to‐severe AD exhibit Th2‐skewed serum biomarkers together with differentially elevated Th1‐, Th17‐ and Th22‐type cytokines/chemokines, and that the underlying pathogenic biological mechanisms vary among patients [19]. Consequently, personalized therapy that targets the unique immune dysfunction characteristics of each individual AD patient may result in maximal clinical improvement.

This study revealed that A‐AHT significantly decreased the levels of serum IL‐2, IL‐6, IL‐10, TNF‐α, and IFN‐γ in all patients with moderate‐to‐severe atopic dermatitis (AD). Furthermore, after a 7‐week treatment period, the A‐AHT group exhibited a notable reduction in serum total IgE concentration. Similarly, a significant decrease in serum total IgE levels was observed in the Dupilumab group, with no statistically significant difference compared to the A‐AHT group. However, it is worth noting that in this study, among the enrolled child and adolescent patients with AD, Dupilumab treatment did not significantly improve the levels of these serum cytokines. The reason for the withdrawal of randomized adult patients in the Dupilumab group from the study was the high pricing ($2780.8/300 mg, $2038.76/200 mg). Overall, the efficacy of A‐AHT in the subgroup of infant and adolescent patients with AD was comparable to that observed in the broader study population. However, it is noteworthy that serum IgE levels did not exhibit a significant improvement at week 8 compared to baseline. Additionally, in one child patient who demonstrated notable clinical improvement after eight treatments, a slight elevation in serum IgE was observed. It is crucial to acknowledge that IgE levels can fluctuate tremendously due to food allergy throughout childhood. However, immunotherapy outcomes in children are not reliably reflected by IgE levels when clinical manifestations are not taken into account [20].

The primary scientific limitation preventing the acceptance of A‐AHT as a standard treatment for atopic dermatitis (AD) among physicians is the insufficient understanding of its therapeutic mechanism of action. The systemic immunomodulatory effects observed cannot be solely attributed to the local inflammation reaction at the injected acupoints [21]. The present results suggest that the suppression of hyperactive cells and their cytokines in AD including regulatory T cells producing IL‐10, Th1 cells producing IFN‐γ and TNF‐α, as well as Th2 cells producing IL‐2 and IL‐6 which were induced by A‐AHT, provided a systemic anti‐inflammatory and immunomodulatory effect. We hypothesize that the patient's autologous blood injection functions as an individualized, multicomponent vaccine‐like stimulus. The inflammation response at the injected acupoints may elicit a local immune reaction. Although these blood constituents are “self” within the vessels, they may be perceived as “non‐self” in muscle tissue. Under this premise, injected blood may act as autoantigens and elicit an autoimmune response. After repeated administrations, the antigenic determinants might be unmasked and subsequently recognized by the host. This approach elicits host immune responses directed at AD‐relevant components and is associated with increased neutralizing mediators, induction of immune tolerance, and re‐establishment of a new immune homeostasis [7, 10, 22]. Furthermore, our recent animal studies indicate that the potential mechanism of A‐AHT may also involve sustained stimulation of the acupoints. The anti‐inflammatory and immune‐regulatory effects of acupoint stimulation may be determined by acupoint specificity. Guided by the principles of acupoint selection in Traditional Chinese Medicine (TCM) theory, ST36, SP10, and LI11 are the most frequently used acupoint combinations for AD treatment [23]. These acupoints are believed to activate qi and blood circulation and regulate immune function [9, 24, 25]. Our recent study has demonstrated that A‐AHT significantly upregulates FoxP3 + CD4 + T cells in murine spleen, as well as enhances FoxP3 expression in dorsal skin lesions of DNCB‐induced atopic dermatitis in Balb/c mice. These results suggest that this treatment may hold potential for activating Treg cells [10]. Therefore, further studies are required to evaluate the activation of Treg cells in clinical trials to identify the immunomodulatory mechanism of A‐AHT.

This clinical trial has several limitations, including relatively small number of patients (especially in the dupilumab group), significant difference in mean age between study groups, and short intervention duration.

Further randomized, double‐blind, controlled clinical trials with larger sample sizes (e.g., at least 50–60 participants, with 25–30 patients per arm in the A‐AHT and placebo or comparator groups), a longer intervention period (e.g., 16 weeks), and extended follow‐up are needed to better define the clinical utility of A‐AHT for atopic dermatitis. As the primary efficacy endpoint, the study should use the response rate at week 16 for achieving Eczema Area and Severity Index (EASI‐75) and/or an Investigator Global Assessment (IGA) score of 0–1 [26], with SCORAD and patient‐reported outcomes included as supportive measures. Immunomodulatory evaluations may be streamlined to a focused biomarker panel (e.g., total IgE, eosinophil count, lactate dehydrogenase (LDH), IL‐10, IFN‐γ, and TNF‐α) as secondary outcomes.

## 5 Conclusions

In conclusion, our preliminary findings suggest that A‐AHT improved clinical outcomes in patients with moderate‐severe AD and exerted systemic immunomodulatory effects, without significant side effects. Additional studies are warranted to determine the clinical utility of this intervention in AD.

## Author Contributions

All authors played a significant role in the study, contributing to various aspects such as conceptualization, research design, implementation, data collection, analysis, and interpretation. Dong‐shu Zhang conceived and drafted this article. Yu‐ling Wang, Ying‐qi Lin, Xing‐ru Yuan, Xin‐yan Li, and Shu‐ping Liang collected clinical data. Xuan Li and Xin‐ru Zhang interpreted the data, composed the article, and prepared the tables and figures. Ying Wu, Zhi‐qian Huang, and Yong Chen conducted statistical analysis.

## Ethics Statement

Ethics approval for this study was granted by the Ethics Committee of the Affiliated Dongguan People's Hospital of Southern Medical University, and all participants provided written informed consent.

## Conflicts of Interest

The authors declare no conflicts of interest.

## AI Use Statement

ChatGPT was used only for language editing and grammar correction. It was not used to generate scientific content, interpret data, or draw conclusions. The authors reviewed and approved the final manuscript and take full responsibility for its content.

## Supporting information

Supporting File

## Data Availability

Raw data supporting the conclusions are available from the authors and can be shared without undue restriction.
