# Supplementary material for: Efficacy, Safety and Immunomodulatory Effect of Intramuscular Injections of Autologous Whole Blood Into Acupoints in Patients With Atopic Dermatitis: A Randomized Controlled Trial
Source: Immun Inflamm Dis. 2026 Apr 20;14(4):e70453. doi: 10.1002/iid3.70453 (PMC13096715; doi:10.1002/iid3.70453)
Supplement: Supplementary file 1 — Supporting File [file IID3-14-e70453-s001.pdf]

Supplementary Table S1. Dosage of Dupilumab determined based on the age and weight of infants and adolescents

**Age/Weight Range**	**< 6 years old**		**> 6 years old**	
	**First dose**	**Subsequent doses**	**First dose**	**Subsequent doses**
5-15kg	200mg	200mgQ4W	—	
15-30kg	300mg	300mgQ4W	600mg	300mgQ4W
30-60kg	—		400mg	200mgQ2W
>60kg			600mg	300mgQ2W
